# Superparamagnetic polyhemoglobin-tyrosinase nanocapsules: a novel biotherapeutic with enhanced tumor suppression with control by external magnetic field

**DOI:** 10.3389/fbioe.2025.1562145

**Published:** 2025-04-30

**Authors:** ChenHui Zhao, Thomas Ming Swi Chang

**Affiliations:** Artificial Cells and Organs Research Centre, Departments of Physiology, Medicine and Biomedical Engineering, Faculty of Medicine and Health Sciences, McGill University, Montreal, QC, Canada

**Keywords:** nanocapsules, artificial cells, nanobiotherapeutic, nanobiotechnology, drug delivery, drug targeting, cancers, superparamagnetic

## Abstract

**Introduction:**

Our recent study shows nanobiotherepeutic Polyhemoglobin-Tyrosinase-Nanocapsules (PolyHb-Tyr-Nano) have strong anti-tumor abilities in multiple cancer lines. However, despite their tumor inhibitory potential, some internal tumor sites can be difficult to reach.

**Methods:**

In this paper, based on Chang’s original finding that artificial cells containing magnetic material can be controlled by external magnetic fields, using nanoprecipitation methods, we modified this biotechnological nanotherapeutic with superparamagnetic properties, which shown to be attracted and guided by external magnets.

**Results:**

By fluorescence microscopy, we show that external magnetic field improved the local deposition of the nanorobotic superparamagnetic PolyHb-Tyr-nano at the tumor microenvironment (TME), significantly preventing their clearance, to stay at the tumor site despite repeated washings. This allowed time for them to enter the tumor cells to act intracellularly. In cell proliferation tests and tumor migration study, their tumor inhibitory action on the four cancer cell lines: Hepa 1-6 liver cancer line, A549 lung cancer line, HeLa cervical cancer line, and MCF7 breast cancer line are also retained effective, a low cell viability and tumor migration was observed. Furthermore, the addition of superparamagnetic property has enhanced the nanocapsules uptake and tumor inhibitory abilities, significantly improved their drug effect on tumor cells. Via cell viability test, PAL assay, oxidative stress detection, and mitochondria membrane potential studies, the PolyHb-Tyr-nano has shown improved tumor killing, by amino acid reduction, reactive oxygen species (ROS) generation, to mitochondria activity reduction in the presence of external magnetic fields.

**Discussion:**

Our results showed the efficacy of the nanorobotic superparamagnetic PolyHb-Tyr-nano on anti-tumor effect in multiple cancer lines. This novel nanobiotherapeutic has the potential for future cancer therapy, and can enhance drug localization, targeted delivery, and combination therapies.

## Graphical Abstract

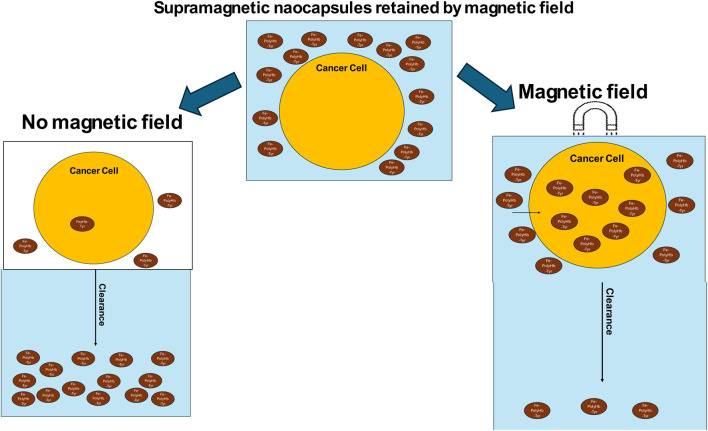

## 1 Introduction

**FIGURE 1:**
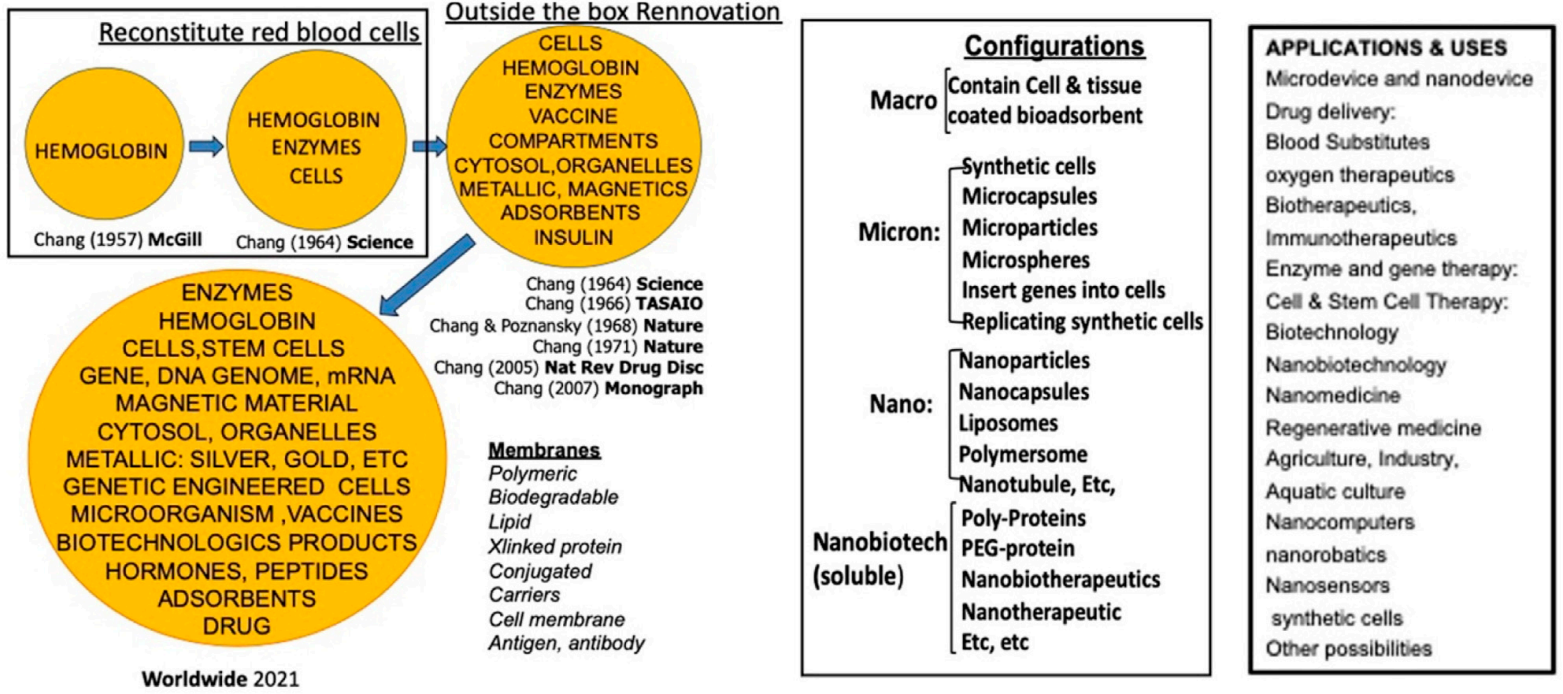
Historical development and different configurations and applications of artificial cells. From (Chang 2019)^1^ with copyright permission from author and Taylor and Francis publisher.

In 1964, Chang introduced the concept of “artificial cells”, which involves bioencapsulation of biological materials like hemoglobin and enzymes within ultrathin polymer membranes to replace or replenish missing cellular functions (Chang, 2019). This innovative approach led to various applications and configurations, including artificial cells of micro and nanodimensions, nanoparticles, nanocapsules, liposomes, nanobiotherapeutics and others (Figure 1) (Chang, 2005; Prakash et al., 2007). In the field of cancer research, microcapsules, nanocapsules, and liposomes are advanced drug delivery systems to improve the safety, bioavailability and targeting of therapeutic agents. The outer coatings can be polymeric, e.g., biodegradable polylactic acid (PLA), or lipid-based phospholipids, with enhanced drug solubility. Along with various surface markers and ligands, these tiny artificial cells can allow a controlled drug release, reduce side effects and immunological reactions. Chang reported the first successful use of bioencapsulated asparaginase to deplete asparagine and thus suppress the growth of lymphosarcoma in mice model (Chang, 1971).

Besides the asparaginase depletion as cancer therapy. Chang’s team introduced a soluble Polyhemoglobin-Tyrosinase (PolyHb-Tyr) complex and nanoencapsulated this to form PolyHb-Tyr-nanocapsules (PolyHb-Tyr-nano) (Yu and Swi Chang, 2004; Wang and Chang, 2012). The rationale behind it was that melanoma requires the extracellular supply of substantial amount of tyrosine. Thus, tyrosinase has been used successfully to deplete the extracellular supply of tyrosine to melanoma in a mice model, suppressed its growth and demonstrated safety in vital organs (Wang and Chang, 2021). However, other cancer cell lines do not require external supply of tyrosine since they do not require large amount of tyrosine and can synthetize the needed tyrosine intracellularly. We therefore try to see if intracellularly located nanocapsules containing tyrosinase can remove the intracellularly synthetized tyrosine to suppress the growth of these cancer cell lines. The result showed that PolyHb-Tyr-nano acted as an amino acid depletion therapy, causing significant tumor reduction in the four tested cancer lines.

The significance and advantage of nanocapsules are accompanied by their limitations. It has always been a goal to achieve a safe, non-invasive, and precise targeting to the internal tumor sites. In order to enhance the ability of nanobiotherapeutic PolyHb-Tyr-nano to target and retain at the desired internal tumor sites, we have developed the nanorobotic biocompatible Fe_3_O_4_ superparamagnetic PolyHb-Tyr-nano. Superparamagnetism is a unique magnetic property present in nanoparticles. When the thermal energy is sufficient to randomize the magnetic moments of particles, they will have the ability to respond to magnetic fields without retaining magnetization.

This is based on Chang’s original finding that artificial cells containing magnetic material can be controlled by external magnetic fields (Chang, 1966), which now is being actively explored for cancer therapy (Wang and Chang, 2023). By using external magnetic field, the artificial cell nanocapsules can be guided and retained at the tumor sites, facilitating its deposition and cell entry. Offering advantages over traditional targeting methods based on passive EPR effects or receptor-specific modifications (Chehelgerdi et al., 2023). It is rather low-cost and convenient development compared to active targeting, also can be very specific compared to passive targeting.

In this present study, we have successfully constructed the superparamagnetic Fe_3_O_4_ PolyHb-Tyr-nano and showed that the addition of superparamagnetic property does not influence PolyHb-Tyr-nano antitumor effect on cancer lines. We have demonstrated that by applying external magnetic field using simple magnets can effectively retain superparamagnetic-PolyHb-Tyr-nano after consecutive clearance, and this facilitates nanocapsules cell uptake. Consequently, significantly improved the drug effect on tumor cells, from amino acid reduction, reactive oxygen species (ROS) generation, to mitochondria activity reduction. Furthermore, superparamagnetic PolyHb-Tyr-nano has high biocompatibility for the following reasons: the nanocapsule membrane is biodegradable, as the outer coating of PLA biodegrades and hydrolyses into carbon dioxide and water (Angelin Swetha et al., 2023; Luo et al., 2019). The PolyHb has been carried out in our laboratories for extensive studies and analysis for its stability and safety to use, its different properties are shown in Table 1. (Hoq and Chang, 2023). The Fe_3_O_4_, PolyHb and tyrosinase biodegrade into their essential components, either enters the bodies iron pool, or are eliminated from cells. All the results indicate the potentials for superparamagnetic PolyHb-Tyr-nano on targeted cancer therapy, by cooperation with external magnetic fields, its efficacy supports further investigations in animal studies.

**TABLE 1:** Detailed analysis of PolyHb has been carried out in our laboratory including different properties as shown in the table. Table adapted from Hoq and Chang (2023), licensed under CC BY.

P_50_	Hb Conc.	COP	Viscosity	MW	Hill coefficient, n50	MetHb content	K autoxidation (rate constant of autoxidation)	K NO (second-order rate constant of NO oxidation)
26.2 ± 0.2 mmHg	5 g/dL	20 mmHg	3 cP	>250 kDa	2.54 ± 0.03^19^	5.12% ± 1.67%	In the presence of superoxide and hydrogen peroxide, PolyHb shows oxidation of Hb (Fe^2+^) to metHb (Fe^3+^). This was prevented when PolyHb contains crosslinked antioxidant enzymes.	NO is important in maintaining the normal dilation of the microcirculation. Our present study studies the free suspension of hepatocytes. There is no microcirculation in the free suspension of hepatocytes, and thus, we did not follow this for the present study.

## 2 Results

### 2.1 Construction of superparamagnetic-Fe_3_O_4_-PolyHb-Tyr-nanocapsules

**FIGURE 2:**
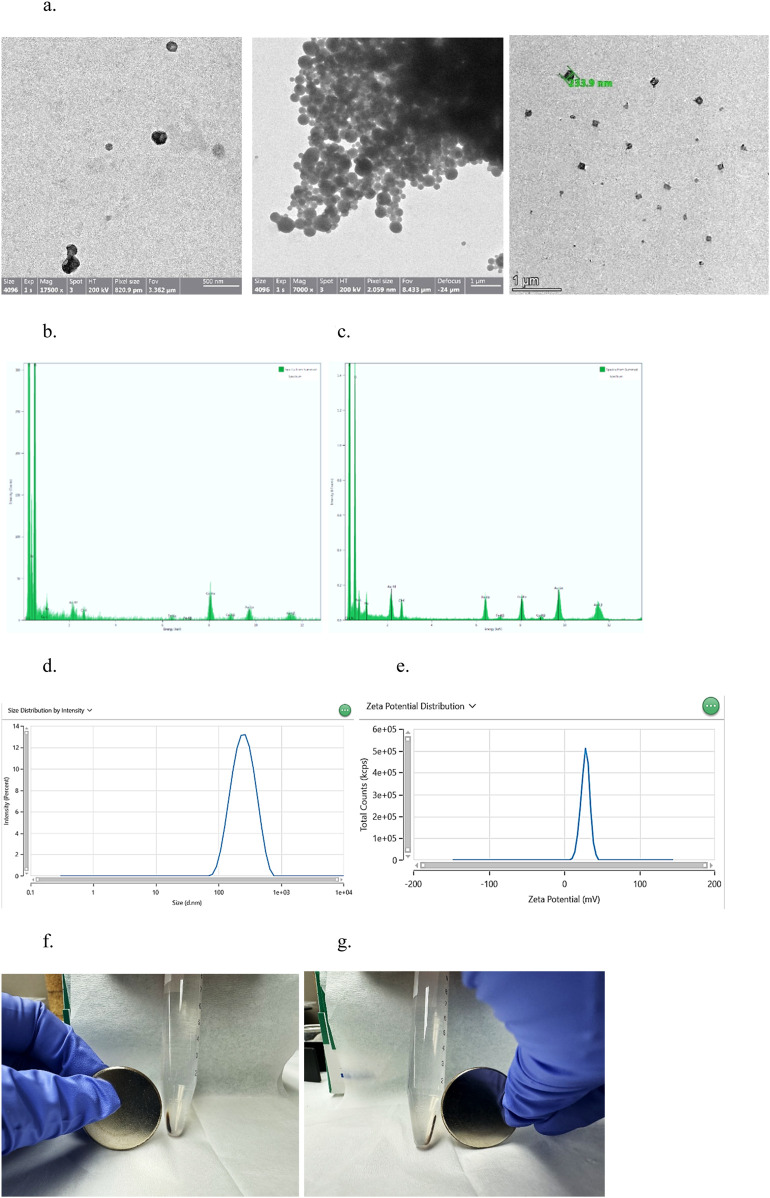
results indicating the successful formation of superparamagnetic nanocapsules. **(a)** TEM photo of Superparamagnetic (left, middle) and PolyHb-Tyr-artificial cell nanocapsules (right) around 233.9 nm, at magnification of 7000x and 1,750,00x. **(b)** EDS analysis of surface composition of PolyHb-Tyr-artificial cell nanocapsules. **(c)** EDS analysis of surface composition of magnetic artificial cell nanocapsules. **(d)** DLS distribution graph showing the average size of the superparamagnetic nanocapsules. **(e)** Zeta potential distribution graph showing the average zeta potential of the superparamagnetic nanocapsules. **(f,g)** Showing the magnetic-polyHb-tyr-PLA-nanocapsules being attracted to the magnet on left/right sides of the test tube.

Superparamagnetic nanocapsules are based on nanoencapsulation of magnetic nanoparticles with biologics to allow for “robotic” control after injection (Chang, 1966; Wang and Chang, 2023). Using the nanoprecipitation method, superparamagnetic-Fe_3_O_4_-PolyHb-Tyr-nanocapsules were formed, its spherical shape and size were confirmed by transmission electron microscopy (TEM) (Figure 2a). Energy Dispersive X-ray Spectroscopy (EDS) analysis showed that the major components of PolyHb-Tyr-nano are carbon and contain few iron Fe ions (Figure 2b). Whereas the major components of superparamagnetic PolyHb-Tyr-nano consist of carbon membrane and contain large amounts of iron Fe ions. Suggesting successful production of superparamagnetic artificial cells containing PolyHb-Tyr (Figure 2c). Dynamic Light Scattering (DLS) testings has shown its Z-average size to be 224 nm and a polydispersity index of 0.1748 (Figure 2d). An average zeta-potential of 27.97 mV and conductivity of 0.081 mS/cm to show its stability and property (Figure 2e). By holding a small N52 grade 14,400 G neodymium disc magnet around the test tube, the superparamagnetic PolyHb-Tyr-nano are attracted to the magnet, either on the left or right sides of the test tube (Figures 2f, g).

### 2.2 Superparamagnetic property does not influence anti-tumor ability of PolyHb-Tyr-nano

#### 2.2.1 Proliferation assay

Multiple cancer lines were included in the study: Mice liver cancer Hepa 1-6 line, human lung cancer A549 line, cervical cancer HeLa line, and breast cancer MCF7 line. Proliferation assay was performed to determine the treatment effects on cell proliferation. With n = 5 on the 4 cancer cell lines, and statistically tested by one-way ANOVA with p < 0.05.

**FIGURE 3:**
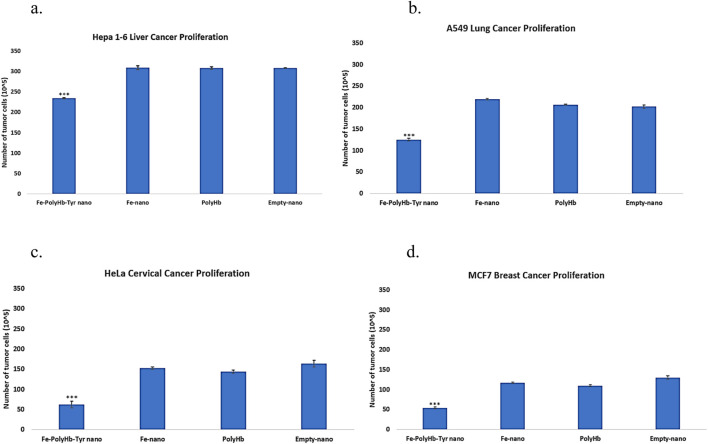
Total cell counts at 48 h representing the proliferation of cancer lines, treatment groups are as follows: Superparamagnetic PolyHb-Tyrosinase nanocapsules (Fe-PolyHb-Tyr-nano), Fe_3_O_4_ nanocapsules (Fe-nano), PolyHemoglobin (PolyHb), Empty Polylactic acid nanocapsules (Empty-nano). **(a–d)** The proliferation trend showing number of cells at 48 h under different treatments for Hepa 1-6, A549, HeLa, and MCF7 cancer lines.

The superparamagnetic PolyHb-Tyr-nano showed significant effects on reducing cell proliferation (Figures 3a–d). The superparamagnetic nanocapsules without PolyHb-Tyr were known as Fe-nano group, had shown no significant difference from the control group, and proved the feasibility of Fe_3_O_4_ in human cells. The superparamagnetic PolyHb-Tyr-nano had significantly lower cell proliferation than all other groups: Fe-nano, PolyHb and empty nanocapsules.

#### 2.2.2 Scratch assay

**FIGURE 4:**
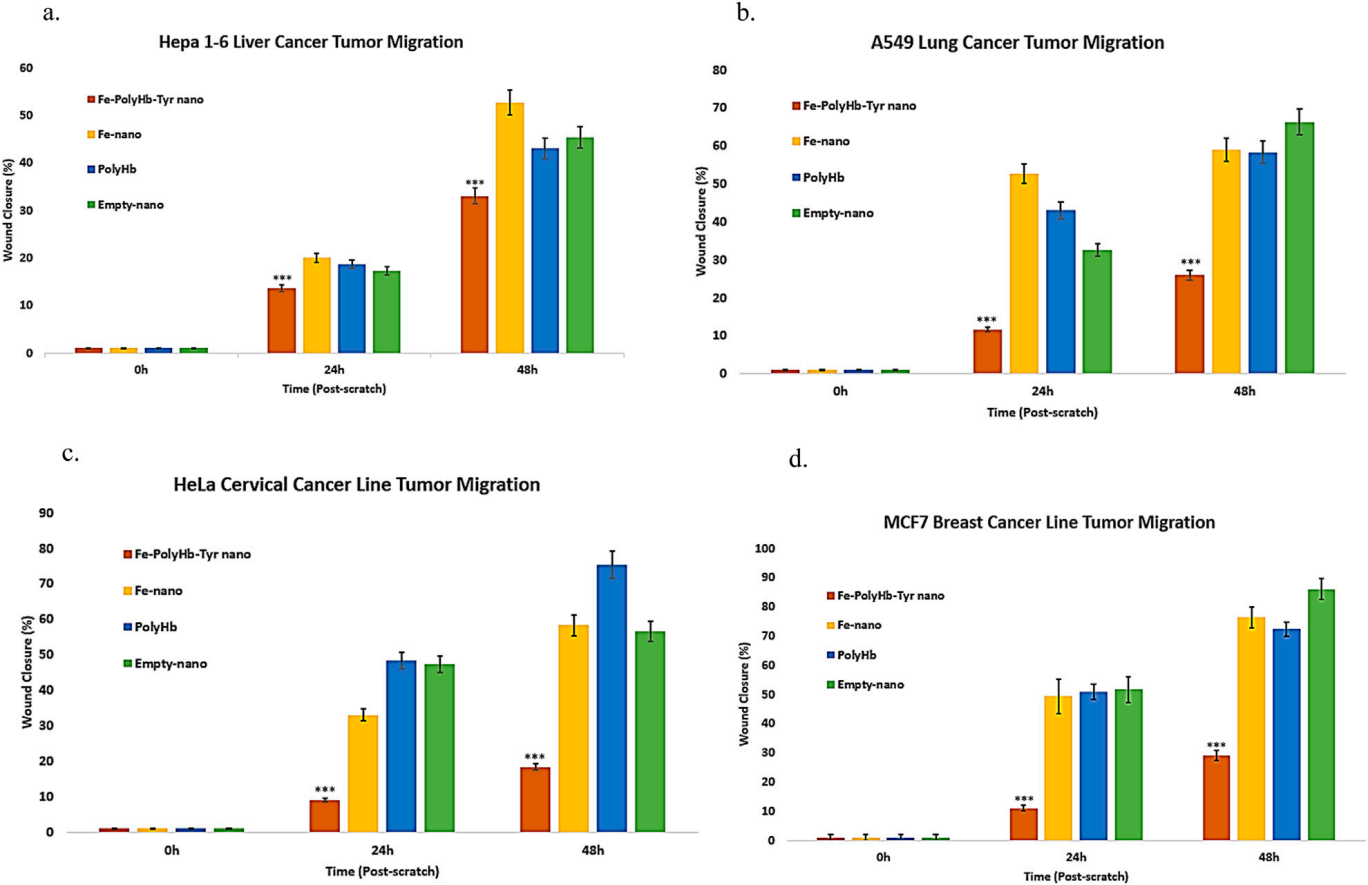
Scratch assay lasting for 48 h, treatment groups are as follows: Superparamagnetic PolyHb-Tyrosinase nanocapsules (Fe-PolyHb-Tyr-nano), Fe_3_O_4_ nanocapsules (Fe-nano), PolyHemoglobin (PolyHb), Empty Polylactic acid nanocapsules (Empty-nano). **(a–d)** Tumor migration vs. time graph for Hepa 1–6 liver, A549 lung, HeLa cervical, and MCF7 breast cancer lines over 48 h.

Scratch assay was performed on a 48 h period to determine the different treatment effects on tumor migration and metastasis, under low FBS medium to minimize tumor proliferation. The scratched wound distances before and after tumor migration were measured by ImageJ. The percentage of tumor migration was determined for each treatment. The results showed that superparamagnetic-PolyHb-Tyr-nano was effective with significantly lower tumor migration compared to other groups at all times: Fe-nano, PolyHb, and Empty-nano groups. In the liver cancer line (Figure 4a), lung cancer line (Figure 4b), cervical cancer line (Figure 4c), and breast cancer line (Figure 4d).

### 2.3 External magnetic field increase superparamagnetic-PolyHb-Tyr-nano deposition, cell entry, and tumor inhibition

#### 2.3.1 External magnetic field effect on superparamagnetic Polyhb-Tyr-nano localization

**FIGURE 5:**
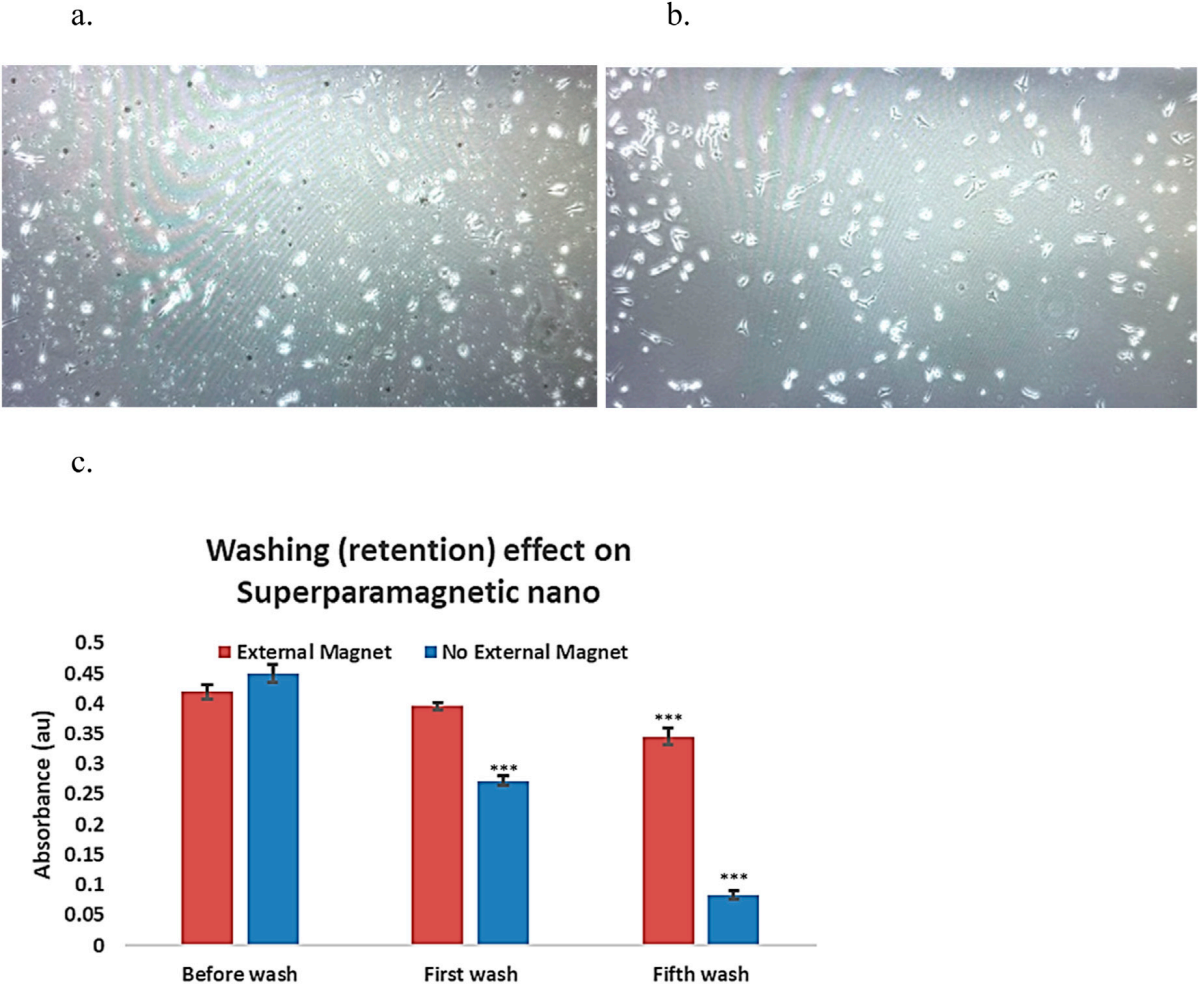
External magnetic affect on superparamagnetic-nanocapsules localization photos using invereted microscopy ×20 magnification, and the retention effect in culture plates. **(a)** Superpramagnetic-PolyHb-Tyr-nano group, after 5 washes, in presence of external magnetic-field, lots of nanocapsules (small particles) are present in the cell culture. **(b)** Superparamagnetic-PolyHb-Tyr-nano group, after 5 washes, with NO external magnetic-field, much less nanocapsules (small particles) are present in the cell culture. **(c)** Superparamagnetic nanocapsules supernatent, with or without magnetic field, supernatant were taken before wash, at first wash, and at fifth wash.

Five consecutive washes were applied to remove nanocapsules, mimicking the body clearance. With an external magnetic field, the superparamagnetic nanocapsules are prevented from clearance (Figure 5a). Compared to those without external magnetic fields, most superparamagnetic nanocapsules were washed away from the plate (Figure 5b).

To assess the impact of external magnets on superparamagnetic nanocapsule localization. Supernatant from the superparamagnetic-PolyHb-Tyr-nano treatment groups was taken after washings, with and without external magnetic field, then compared their absorbance at 500 nm for PolyHb content with n = 3 (Figure 5c). Both external magnetic and non-magnetic groups had similar absorbance of 0.42 and 0.451 before washing. After the first wash, the external magnetic group had higher absorbance compared to the non-external magnetic group (0.396 vs. 0.272). After the fifth wash, the external magnetic group had significantly higher absorbance compared to the non-external magnetic group (0.346 vs. 0.084). The results showed with consecutive washings to mimic the body clearance, the external magnet can attract and retain the superparamagnetic nanocapsules locally and not be washed off. Therefore, the absorbance in the external magnetic group is significantly higher, suggesting higher nanocapsules concentration locally than the non-external magnetic field group.

#### 2.3.2 External magnetic field effect on tumor cell viability

To test the effect of an external magnetic field on superparamagnetic PolyHb-Tyr-nano, magnets were placed outside the cell culture dish to retain the nanocapsules during five consecutive washes, results were compared with the non-magnetic group.

**FIGURE 6:**
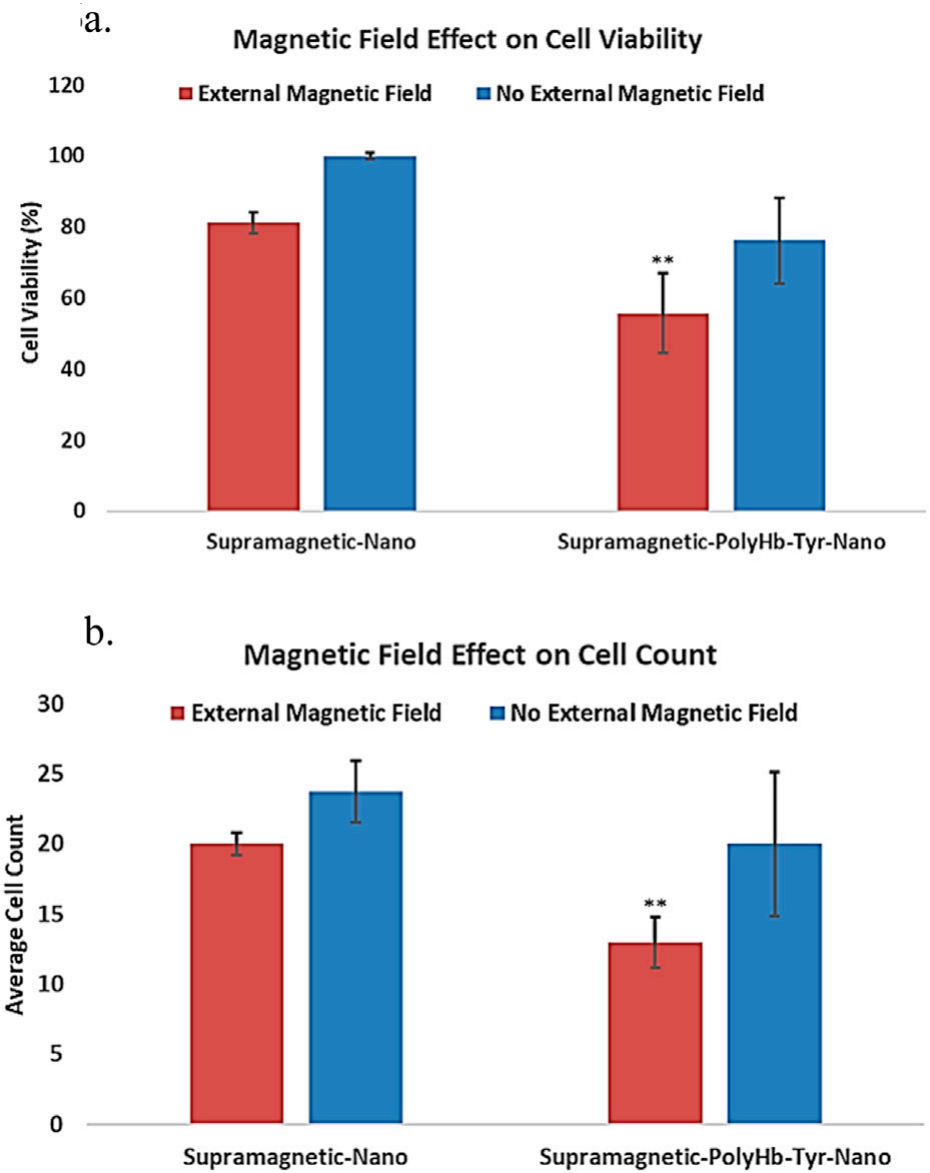
under external magnetic field, the effect of superparamagnetic nanocapsules. Control superparamagnetic-nano = supramagnetic-nano, superparamagnetic polyhemoglobin-tyrosinase nanocapsule = superamagnetic-PolyHb-Tyr-nano. Red bar = external magnetic field. Blue bar = non-magnet substitute. **(a)** cell viability of Hepa 1-6 using superparamagnetic nanocapsules with or without external magnetic field. **(b)** average cell counts of Hepa 1-6 using superparamagnetic nanocapsules with or without external magnetic field.

Superparamagnetic-PolyHb-Tyr-nano were attracted to the external magnetic field, thus stayed in the cell culture dish during 5 times cell wash. With an external magnet, cell viability in the superparamagnetic-PolyHb-Tyr treated group was 55%, compared to 76% without the external magnetic field (p < 0.05) (Figure 6a). In the absence of an external magnetic field, some nanocapsules could still stay in the tumor microenvironment, attached to the cell surface or entered the cells. The result suggests a significant effect of external magnetic field on retaining superparamagnetic-PolyHb-Tyr-nano, and its effect on cells.

The total cell counts results have the same trend as cell viability, lowest cell counts in superparamagnetic PolyHb-Tyr-nano group in the presence of an external magnet, further supports the result of the cell viability study (Figure 6b). Overall, the results suggest that the addition of external magnetic field can significantly help in retaining nanocapsules at local tumor sites despite repeated washings. This facilitates the entry of nanocapsules into the cells and significantly enhances the anti-tumor effect.

#### 2.3.3 External magnetic field effect on tumor cell amino acid levels

**FIGURE 7:**
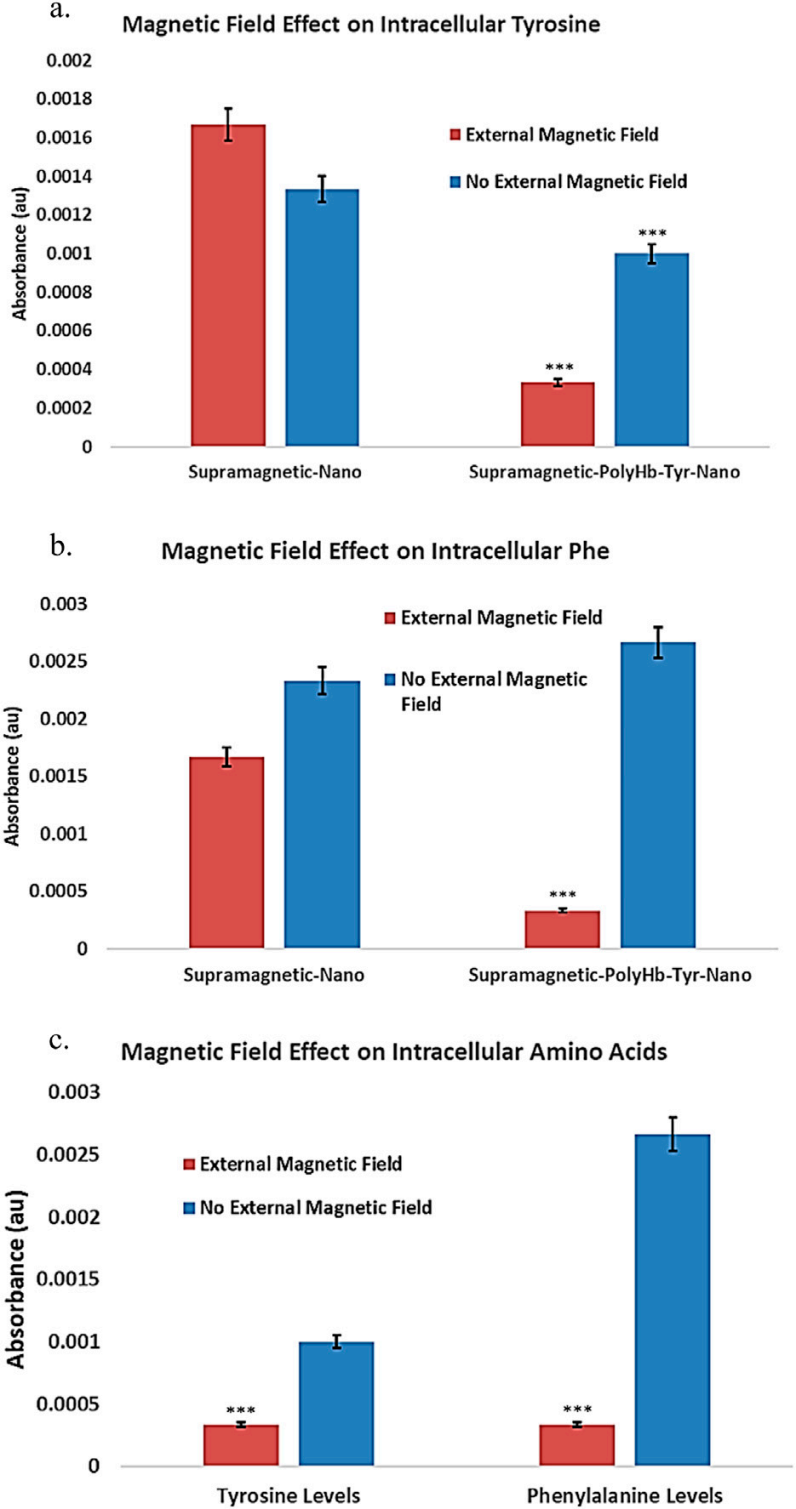
Under external magnetic field, the effect of superparamagnetic nanocapsules on intra and extracellular amino acid tyrosine (Tyr) and phenylalanine (Phe) levels. Control superparamagnetic-nano = supramagnetic-nano, superparamagnetic polyhemoglobin-tyrosinase nanocapsule = superamagnetic-PolyHb-Tyr-nano. Red bar = external magnetic field. Blue bar = non-magnet substitute. **(a)**: intracellular tyrosine of Hepa 1-6 using superparamagnetic nanocapsules with or without external magnetic fields. **(b)**: intracellular phenylalanine of Hepa 1-6 using superparamagnetic nanocapsules with or without external magnetic fields. **(c)**: Direct comparison of intracellular amino acid levels with external magnetic field presence in the superparamagnetic-PolyHb-Tyr-nano group.

The main function of PolyHb-Tyr is to reduce intracellular tyrosine amino acid levels and establish amino acid depletion, limiting tumor nutrient sources to inhibit tumor growth. The intracellular Tyrosine (315 nm) (Figure 7a) and its upstream amino acid phenylalanine (290 nm) (Figure 7b) were detected by PAL enzyme assay. The results showed that in the presence of an external magnetic field, the superparamagnetic PolyHb-Tyr-nano stayed in the tumor microenvironment and entered tumor cells, to deplete their internal amino acids. Therefore, they have significant lower amino acid levels compared to without external magnetic field group (Figures 7a–c).

#### 2.3.4 External magnetic field effect on nanocapsule entry

**FIGURE 8:**
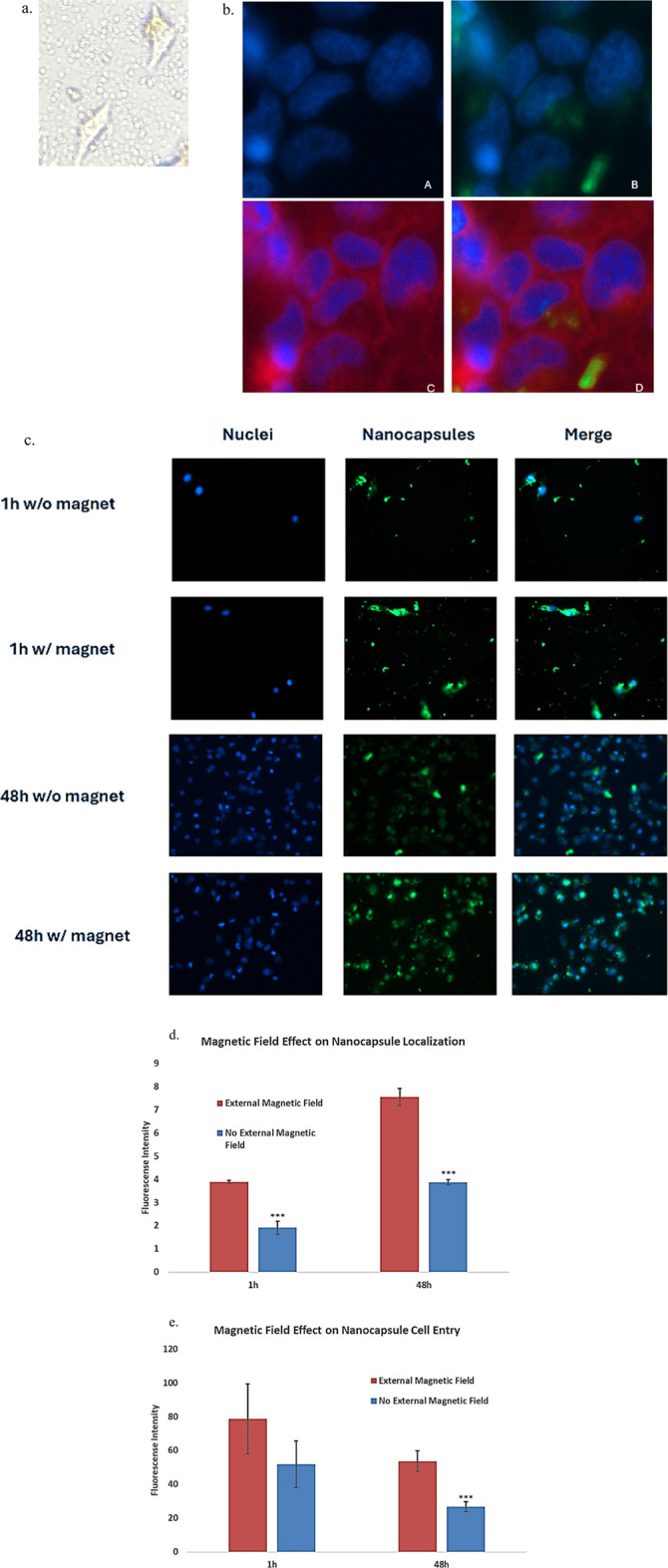
To show that the nanocapsules are able to enter the Hepa1-6 cells and not simply accumulate in the tumor microenvironment, the entry of nanocapsules into cells was confirmed using fluorescence microscopy under the help from microscopy facility professionals. Fluorescent dyes were applied as follows: Hoechst 33,342 (blue fluorescence) for cells, Coumarin 6 (green fluorescence) for nanocapsules, and red plasma membrane dye for cell membrane staining. Photos were taken on a timely fashion showing Fe-PolyHb-Tyr-nanocapsules entrance into the cells using Axiovert 1 fluorescence microscope at ×20 objectives. **(a)** Inverted microscopy showing non-dyed cells and nanocapsules, both co-localizing with the cell and in the tumor microenvironment. **(b) Panel (a)** the cell nucleus stained with DAPI showing blue fluorescence. Panel **(b)** Co-localization of the blue fluorescent cell nucleus and green fluorescent nanocapsules in the cytoplasm, and of green fluorescence surrounding the nucleus, showing nanocapsules that entered the cell but stayed in the cytoplasm instead of entering the cell nucleus. **Panel (c)** Red plasma membrane dye marks the border of cells. **Panel (d)** The addition of the plasma membrane dye confirming the cell entry of green nanocapsules inside the cytoplasm, around cell nucleus. Also showing some nanocapsules inside the tumor microenvironment. **(c)**: photos taken on a timely fashion, showing on the 1^st^ hour and 48^th^ hour of cell seeding, with and without external magnet attractions. Showing that the cells proliferate with the fluorescent nanocapsules in their cytoplasm. And the nanocapsules floating in the tumor microenvironment. **(d)**: graph showing the quantified fluorescence intensity from the fluorescence photos, indicating the number of nanocapsules after washes in cell cultures, during 1 h and 48 h, with or without magnetic fields. **(e)**. Graph showing the quantified fluorescence intensity in tumor cells, indicating the number of nanocapsules that entered the cells, during 1 h and 48 h, with or without magnetic fields.

To show the nanocapsules can enter the cells, rather than accumulate in the tumor microenvironment and risking clearance from the body. Inverted microscopy showed the circular nanocapsules surround and co-localize with the cells, but cannot confirm their cell entry (Figure 8a). The entry of nanocapsules into cells was thus confirmed using fluorescence microscopy. Fluorescent dyes were applied, Hoechst 33,342 (blue) for cell nucleus and Coumarin 6 (green) for nanocapsules (Figure 8b). Photographs were taken after incubating the nanocapsules for 1 h and 48 h to demonstrate the successful entry of superparamagnetic PolyHb-Tyr-nanocapsules into cells and their anti-tumor effects with or without external magnetic fields. A red cell plasma membrane dye has stained the cell membranes and marked cell borders, providing clearer visualization of nanocapsules entry (Figure 8b).

Photos showed the green nanocapsules were very close to the blue nucleus, not randomly in the cell-cell junctions, indicating the nanocapsules were taken by the cells (Figure 8b). The addition of red plasma membrane dye marked the borders of the cells, showing the blue nucleus and green nanocapsules were all within the cell membrane, the green and blue colors did not overlap with each other, indicating the nanocapsules were settled in the cytoplasm.

We then visualized the effect of external magnetics. As a comparison, without external magnets, fewer nanocapsules were found in the TME, indicating consecutive washings have cleared nanocapsules from the local TME (Figure 8c). More nanocapsules were observed with the presence of external magnets, this was also quantified by the fluorescence intensity measurement. Higher fluorescence intensity was observed in the presence of external magnetic field (Figure 8d). This suggests that external magnetic fields can help maintain nanocapsules locally after consecutive washes, thus facilitating their cell entry.

At time 1 h, both groups have few blue fluorescence markers, indicating that both groups started with low live cell counts (Figure 8c). At time 48 h, more blue fluorescence signals were observed for the non-external magnet group (Figure 8c). Less blue fluorescence signals were observed for the external magnetic field group. This means that the cell viability is reduced in the presence of an external magnet. Further supports nanocapsule cell entrance and local retention are higher with presence of external magnet, which reduced the cell viability [Figures 8D,E].

#### 2.3.5 External magnetic field on nanobiotherapeutic effects

##### 2.3.5.1 Reactive oxygen species (ROS) generation

**FIGURE 9:**
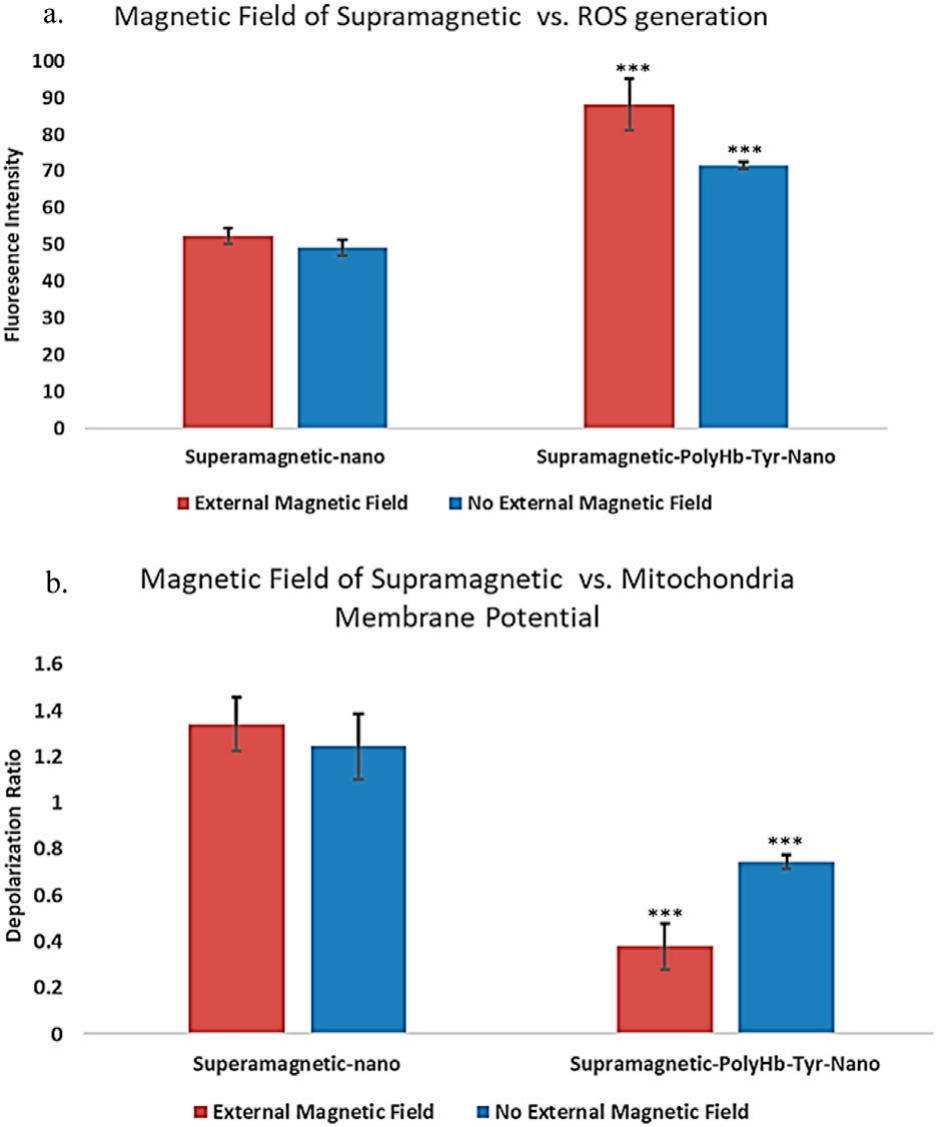
Drug effects of superparamagnetic nanocapsules in Hepa 1-6 liver cancer line with and without external magnetic field. **(a)** ROS fluorescent intensities measurement showing the reactive oxygen species (ROS) generation as a superparamagnetic PolyHb-Tyr-nano drug effect, in the presence and absence of external magnetic fields. Control superparamagnetic-nano = supramagnetic-nano, superparamagnetic polyhemoglobin-tyrosinase nanocapsule = superamagnetic-PolyHb-Tyr-nano. Red bar = external magnetic field. Blue bar = non-magnet substitute. **(b)** Mitochondria membrane depolarization indicates mitochondria activity levels. Showing the membrane depolarization as superparamagnetic PolyHb-Tyr-nano drug effect, in the presence and absence of external magnetic fields.

To show the effects of external magnetic field on superparamagnetic PolyHb-Tyr-nano drug effects in tumor cells, the generation of ROS was tracked with CellROX oxidative stress reagent. This cell-permeable reagent shows strong fluorescence signal upon oxidation that can be measured by fluorescence microscope. As a result, in the presence of external magnetic field, the ROS fluorescent intensity in the superparamagnetic PolyHb-Tyr-nano group was 88.084, and in the control group was 52.367. In the absence of external magnetic field, the ROS fluorescent intensity in the superparamagnetic PolyHb-Tyr-nano group was 71.559, and in the control group was 49.241. P-values <0.05. Thus, the ROS levels are always higher in the PolyHb-Tyr-nano group compared to the control group. This indicates the PolyHb-Tyr can transport oxygen to the hypoxic tumor microenvironment and induce free oxygen radicals that leads to this high ROS. Furthermore, this drug effect/ability to increase cell stress ROS levels can be enhanced with the presence of external magnetic field (Figure 9a).

##### 2.3.5.2 Mitochondria activity assay

Mitochondria is the powerhouse of cells, their activities are correlated with cell metabolism, cell health and various physiological processes. To study the impact of external magnetic field with superparamagnetic PolyHb-Tyr-nano on cells, their effects on mitochondrial activity was also tested. The mitochondrial membrane potential is essential for ATP production and correlated to mitochondria functional state. Thus, the amount of depolarization was determined as an indicator of mitochondria activity level. As a result, in the presence of magnetic field, the red-green fluorescence ratio indicating depolarization of mitochondria membrane potentials, which corresponds to the mitochondria activity level in the superparamagnetic PolyHb-Tyr-nano group was 0.378, and in the control group was 1.339 (Figure 9b). In the absence of magnetic field, the mitochondria activity level in the superparamagnetic PolyHb-Tyr-nano group was 0.745, and in the control group was 1.245. P-values <0.05. Thus, mitochondria membrane potential is always more depolarized in the PolyHb-Tyr-nano group compared to the control group. This indicates a lower mitochondria activity level caused by superparamagnetic PolyHb-Tyr-nanocapsules. Which the PolyHb-Tyr-nanocapsules lead to mitochondria membrane depolarization or mitochondria dysfunction. This drug effect on mitochondria activities can also be enhanced with the presence of external magnetic field.

## 3 Discussion

Artificial cells have developed numerous configurations and can be modified to demonstrate nanorobotic activities, which improves drug-targeted delivery (Chang, 2019) (Figure 1). In this paper, we established the novel nanorobotic superparamagnetic-PolyHb-Tyr nanocapsule artificial cell and validated their efficacy in multiple cancer lines. The modified Fe_3_O_4_ resulted in superparamagnetic property to the nanocapsules (Figure 2) (Chang, 1966), this way, they can act as nanorobots allowing external magnetic field to direct their location, to achieve magnetic-guided localization and enhance tumor site action and retention. From the results, inducing superparamagnetic properties to PolyHb-Tyr-nano does not affect its anti-tumor abilities (Figures 3, 4, 6) and ability to reduce tyrosine levels (Figure 7) over the 48 h observation period in all tested cancer cell lines. Suggesting the induced tyrosine depletion effect is significant to tumor growth. In the later tests, by applying external magnetic field to the cell culture plate, followed by consecutive washings to mimic body clearance, had shown that superparamagnetic PolyHb-Tyr-nano can be controlled by an external magnetic field to stay at the tumor site despite repeated washing of the cell culture (Figures 5, 8). Without external magnetic field, the superparamagnetic PolyHb-Tyr-nano were largely washed away from the TME.

After applying external magnetic fields, the superparamagnetic nanocapsules were retained locally, prevented from clearance. Furthermore, the addition of external magnetic field has enhanced the superparamagnetic PolyHb-Tyr-nano’s drug effects: the nanocapsules localization, cell viability, intracellular amino acid levels, ROS generation, and mitochondria activities are significantly affected than without the magnetic field, due to the concentrated superparamagnetic PolyHb-Tyr-nano cell entry has facilitated to exert stronger drug effects (Figures 6–9). The alternations of the ROS and mitochondria activities suggest the potential pathways of the superparamagnetic PolyHb-Tyr-nano and marks the next step of the study. The study is important because internal organs are difficult to reach. With the nanorobotic superparamagnetic nanocapsules, stronger external magnetics such as MRI may be cooperated to achieve precise drug targeting to tough cancer sites. The external magnets can also be used as follow-up treatments to enhance superparamagnetic nanocapsules localization and prevent them from cleared by the body. This way, the drug bioavailability is significantly increased, which maximize their tumor-inhibitory effects. For superparamagnetic PolyHb-Tyr-nanos, the ability for PolyHb-Tyr-nano retention is critical, because it can both entering the cell and stay within the TME to reduce local amino acids supply that tumor requires for proliferation (Figure 8). Furthermore, external magnet has the potential to precisely control the localization of treatments, therefore the treatment effects stay locally in the tumor area, without effecting other organs or experience global side effects, providing additional layer of safety and biocompatibility of this nanorobotic approach.

Despite these promising magnetic enhancement results, the nanorobotic superparamagnetic therapy is still in its early stages of development. It has lots of potential on nanoencapsulating with other drugs to increase their bioavailability. More combination therapies can also be achieved with superparamagnetic nanocapsules or with PolyHb-Tyrosinase for synergistic effects. Overall, these results showed the potential for targeted, safe, and effective treatment strategies with minimal side effects. Encourage the future experiments to investigate the potential mechanisms of anti-tumor action, move on to more sophisticated tumor models such as 3D culture, organ-on-chips, and/or animal models, using a higher magnetic load, and compare the nanobiotherapeutic effect with commercialized chemotherapy drugs. Once move onto *in-vivo* modes, its LD50 can be studied and compare with the commercial chemotherapeutics doxorubicin in encapsulated form (LD50 of 32 mg/kg) or free doxorubicin (LD50 of 17 mg/kg) (Kanter et al., 1993) and with 5-5 fluorouracil (LD50 in rats are 230 mg/kg, intraperitoneal 70 mg/kg, 450 mL/kg in human) (Cayman chemical, 2023).

## 4 Conclusion

In conclusion, this novel superparamagnetic nanobiotherapeutic PolyHb-Tyr-nano has the potential for further research into future cancer therapy, drug localization, and targeted delivery. Some cancers, especially the metastatic ones are often difficult to treat due to challenges with accessibility. Superparamagnetic nanocapsules can enhance drug localization, cell entry, and amplify drug effects under external magnet guidance. This strategy offers an innovative approach to optimize targeting and enhance bioavailability, emphasizing the potential of nanorobotic superparamagnetic nanobiotechnology in cancer treatment. By future cooperating with other cancer therapies, and modifying with more artificial cell configurations, this nanorobotic approach can provide better targeted, efficient, and safer drug delivery to cancers and other metabolic disease treatments.

## 5 Materials and methods

### 5.1 Materials

Purified ultrapure bovine hemoglobin was purchased from the Biopure company. Hepa1-6, A549, HeLa, and MCF7 cancer lines were purchased from the Creative Biogene company. Glutaraldehyde (25%) (cat# 00376-500) was obtained from Polysciences. Poly(D,L-lactic acid), IV 0.4 dL/g, was obtained from Polysciences Inc. (cat# 16585-10). L-Lysine monohydrochloride (SigmaUltra >99%) (cat# L8662-1 KG), L-tyrosine [98% thin layer chromatography (TLC)] (cat# T2006-1G), and Tyrosinase from mushroom (EC 1.14.18.1, 3,000 units/mg stated activity) were obtained from Sigma-Aldrich (cat# T3824-50KU). Phenylalanine ammonia-lyase (PAL) was obtained from MilliporeSigma Canada Ltd. (cat# P1016-10UN). Bisbenzimide H 33,342 was obtained from Abcam Inc. (cat# ab145597-25 MG). Coumarin 6, 99+%, was obtained from MilliporeSigma Canada Ltd. (cat# 546283-100 MG). CellMask Deep Red Plasma Membrane Stain was obtained from Life Technologies Inc. (cat# C10046). Nutrient agar plates were obtained from Thermo Fisher Scientific (cat# 50948606).

### 5.2 Cell culture

The Hepa 1-6 mice liver cancer line was cultured in standard Dulbecco’s Modified Eagle’s Medium (DMEM) supplemented with 10% fetal bovine serum (FBS). The A549 human lung cancer line was cultured in DMEM/F-12 (Dulbecco’s Modified Eagle Medium/Nutrient Mixture F-12) supplemented with 5% HI FBS. The HeLa human cervical cancer line was cultured in DMEM supplemented with 10% FBS. The MCF7 human breast cancer line was cultured in Minimum Essential Medium (MEM) supplemented with 10% HI FBS 1% non-essential amino acid (NEAA). All cancer lines were cultured under 37°C and 5% CO_2_ in a humidified atmosphere in a HERAcell VIOS 160i CO2 incubator (2021 model with serial no. 42621149).

### 5.3 Preparation of PolyHb-Tyr

Reaction mixtures were prepared containing hemoglobin (10 g/dL) and tyrosinase (6,000 U/mL) in 0.1 M of potassium phosphate buffer, pH 7.6. In the PolyHb mixtures, an equivalent volume of buffer replaced the enzyme. Prior to the start of cross-linking, 1.3 M of lysine was added at a molar ratio of 7:1 lysine/hemoglobin. The cross-linking reaction was started with the addition of glutaraldehyde (5%) at a molar ratio of 16:1 glutaraldehyde/hemoglobin (Yu and Swi Chang, 2004; Wang and Chang, 2012). Glutaraldehyde was added in four equal aliquots over a 15 min period. After 3.5–48 h at 4°C under aerobic conditions with constant stirring, the reaction was stopped with 2.0 M of lysine at a molar ratio of 200:1 lysine/hemoglobin. Solutions were dialyzed in physiological saline solution and passed through a sterile 0.45 μM filter. Aliquots (500 μL) of the 16:1 cross-linked preparation were concentrated using 100 kDa microconcentrators. Samples were centrifuged at 2,500 *g* for 55 min at 23°C, and the retentate was collected. The hemoglobin concentration was determined using cyanomethaemoglobin at 540 nm. We nanoencapsulated the PolyHb-Tyr (38.25 mg/mL, 4500 U Tyrosinase) in the aqueous phase, with 90 mg of PLA considered as the organic phase, following the nanoprecipitation method described previously (Wang and Chang, 2021). The final concentration used as 1X PolyHb-Tyr-nano solution in the viability studies was 6.4 mg/mL + 225 U of tyrosinase.

### 5.4 Preparation of Fe_3_O_4_ poly-Hb-Tyr nanocapsules

The aqueous phase consists of 1 mg/mL Fe_3_O_4_ solution, and 10 mL of PolyHb-Tyr containing 16 μL of tween 20 for the stabilization and aggregation prevention of the nanocapsules. The organic phase consisting of poly-lactic(dl)-acid was injected dropwise into the aqueous phase via a 26G needle, at a rate of 3 mL/min under magnetic stirring at a speed of 6. (Wang and Chang, 2023). The electrostatic Van Der Walls force will allow the formation of the polymeric shell of nanoparticles around PolyHb-Tyr. Stirred at a speed of 6 under 4° ventilation for 1 h to allow full evaporation of the organic solvents, and leave only the PLA nanocapsules in the aqueous phase, forming a 4.5*10^−4^ M concentrated PLA nanocapsule solution. After full evaporation, 150 μL of tween 20 was added to prevent further aggregation and help nanocapsule stabilization. Then, place thin drops of samples onto microscope slides to study the size and morphology of the nanocapsules. All the operations are done under nitrogen to prevent the formation of methemoglobin and protect the enzymes activity. To purify the nanocapsules, centrifuge at 9,000 rpm for 7 min for 3 rounds to isolate the nanocapsules while draining out the free PolyHb-Tyr, the final concentration of the Fe_3_O_4_ nanocapsules was 0.09 mg/mL.

### 5.5 Measure tyrosinase activity

Tyrosinase activity was assessed through the enzymatic reaction of tyrosinase converting tyrosine to form the enzymatic product dopaquinone, which can be measured at 300 nm. The absorbance at 300 nm was followed continuously for 8–14 min using a Perkin Elmer Lambda 4B spectrophotometer, and the changes in optical density per minute were used to analyze the activity of the enzyme (Chang, 1966).

### 5.6 Migration assay

Four cell lines consisting of Hepa1-6, A549, Hela, MCF7. 7 experimental groups (n = 5) including Fe-PolyHb-Tyr-nano, Fe_3_O_4_-nano, PolyHb, Empty-nano, and Blank control group (did not add any drug, only shows cell growth). Seed the cells in 6-well dish, after the cells grow to 80 percent confluence, replace with low-FBS medium to minimize cell proliferation. Create 1 diagonal scratch using a sterile pipette tip, observe under a microscope, and mark spots close to the scratch area. Dilute drug treatments and add 200 μL treatments into each well. Incubate the plate for 24 h and 48 h, wash with PBS, and take photos. Use ImageJ to measure the marked scratch locations and obtain an average percentage of the tumor migration per day ((day1 wound distance - day2 wound distance)/day1 wound distance) *100%.

### 5.7 Tyrosine and phenylalanine concentration measurement

To measure the tyrosine concentration in the intracellular environment, we diluted the sample with 1 mL of DMEM/FBS and centrifuged the cell suspensions at 16,000 x g for 20 min at 4°C to lyse the cells and expose the sample’s intracellular tyrosine. Then, we collected the supernatant in fresh Eppendorf tubes and placed it on ice, discarded the pellet, and performed phenylalanine ammonia-lyase (PAL) enzyme analysis for Tyr and Phe; these are able to convert Tyr to trans-coumarate, which is detectable at 315 nm.

### 5.8 Superparamagnetic external magnetic effect on cells study

Seed Hepa 1-6 liver cancer cells onto 6 well plates (n = 5), allowing cell attachment for 24 h. The next day, add 200 μL of treatments in the middle of the plate, and swirl the plate to well spread the artificial cells. 2 experimental groups were tested: First group: within 10 s of adding treatment, place the magnet under and outside the plate, allow for artificial cell attractions. Second group: within 10 s of after adding treatment, place a plastic (similar size as the magnet, but lack magnetic attraction) under and outside of the plate. After placing the magnet, start to gently change the medium 5 times consecutively (1-2 min each wash) to wash out nanocapsules, with the magnets or substitutes externally. After washing, remove the magnet, swirl the cell culture plate for 1 min to disperse the nanocapsules, and return the plate to the incubator to allow for cell growth for 3 days, with no medium change in between. On day 4, collect and suspend the cells to examine cell viability and total cell counts using a hemocytometer, and using PAL assay to determine intracellular tyrosine levels.

### 5.9 Fluorescence nanocapsules entry study

Preparation of coumarin6 incorporated nanocapsules was done by dissolving coumarin6 into the organic phase solution, together with the dissolved poly-lactic acid, at a dye: PLA ratio of 1:150, followed by nanoprecipitation method described earlier to form Fe_3_O_4_-PolyHb-Tyr-nanocapsules. The fluorescent nanocapsules were added to the cell cultures, followed by consecutive washings with or without external magnets. After incubation, Hoechst dye was added to stain the cell nucleus, (Wang and Chang, 2021; Wang and Chang, 2023), and cell plasma membrane dye was added to mark cell borders. The Hepa1-6 cells were then subjected to Axiovert 1 fluorescence microscopy at ×20 objectives for analysis. The following reflectors were used: 49DAPI for H3258 dye, 10Alexa Fluor 489 for Coum6 dye, and 50 Cy 5 for AF647 dye. The light source intensity was 2%, and the exposure time was set to the standard 150 ms. Photos were taken at 1 h and 48 h to show nanocapsule localization and cell entries, and ImageJ was used to determine fluorescence intensities.

### 5.10 ROS generation study

The generation of ROS was tracked with CellROX oxidative stress reagent. The cells treated with drugs for 1 h and apply external magnetic field, after repetitive washings and a growth period of 48 h, the cells were then incubated with CellROX reagent at 37°C in dark. Then were taken analyzed via fluorescence microscopy, the quantitative fluorescence intensity was measured via ImageJ to compare ROS generation between the groups.

### 5.11 Mitochondria activity assay

The mitochondria membrane potential was determined with JC-1 fluorescence assay, and the fluorescence properties will change depending on the membrane potential. Red fluorescence indicating healthy membrane potential, and green fluorescence indicate membrane depolarization. The cells were initially treated with drugs and external magnetic fields for 1 h, followed by a 48 h growth period, then incubate with JC-1 dyes at 37°C. The fluorescence intensities were quantified and analyzed by ImageJ. The ratio of red-to-green was determined as depolarization ratio.

### 5.12 Statistical analysis

Statistical analysis was performed using Student’s *t*-test or one-way ANOVA, and results were considered significant at a *p*-value of <0.05. Significant level in comparison to the control group: *p*-values are indicated by *<0.05, **<0.01, ***<0.005.

## Data Availability

The raw data supporting the conclusions of this article will be made available by the authors, without undue reservation.
